# Supplementary material for: Reduced biomass burning emissions reconcile conflicting estimates of the post-2006 atmospheric methane budget
Source: Nat Commun. 2017 Dec 20;8:2227. doi: 10.1038/s41467-017-02246-0 (PMC5738352; doi:10.1038/s41467-017-02246-0)
Supplement: Supplementary file 2 — Supplementary Information [file 41467_2017_2246_MOESM2_ESM.pdf]

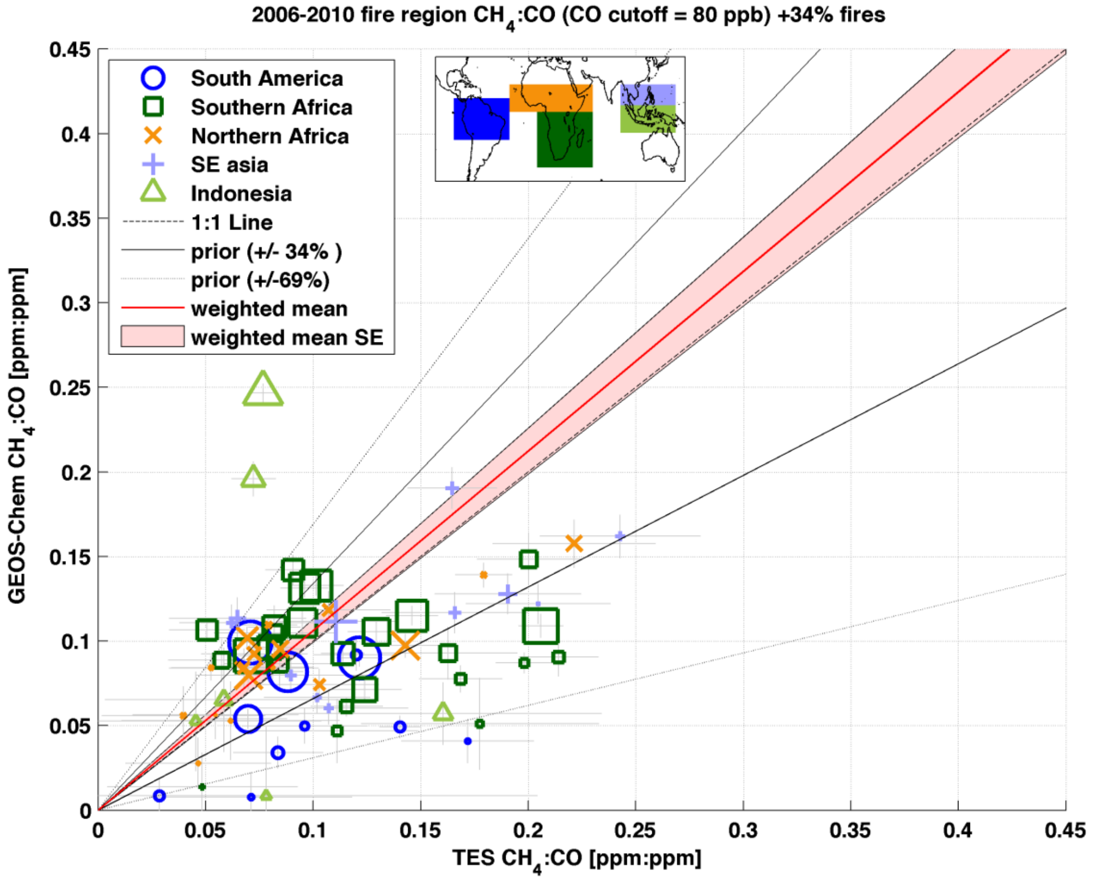

Supplementary Figure 1: Comparison Of CH_4/CO ratios from the GEOS-Chem model and Aura TES data. This figure is the same format as in Figure 6 but the CH_4/CO ratios in the model have been changed by 34%

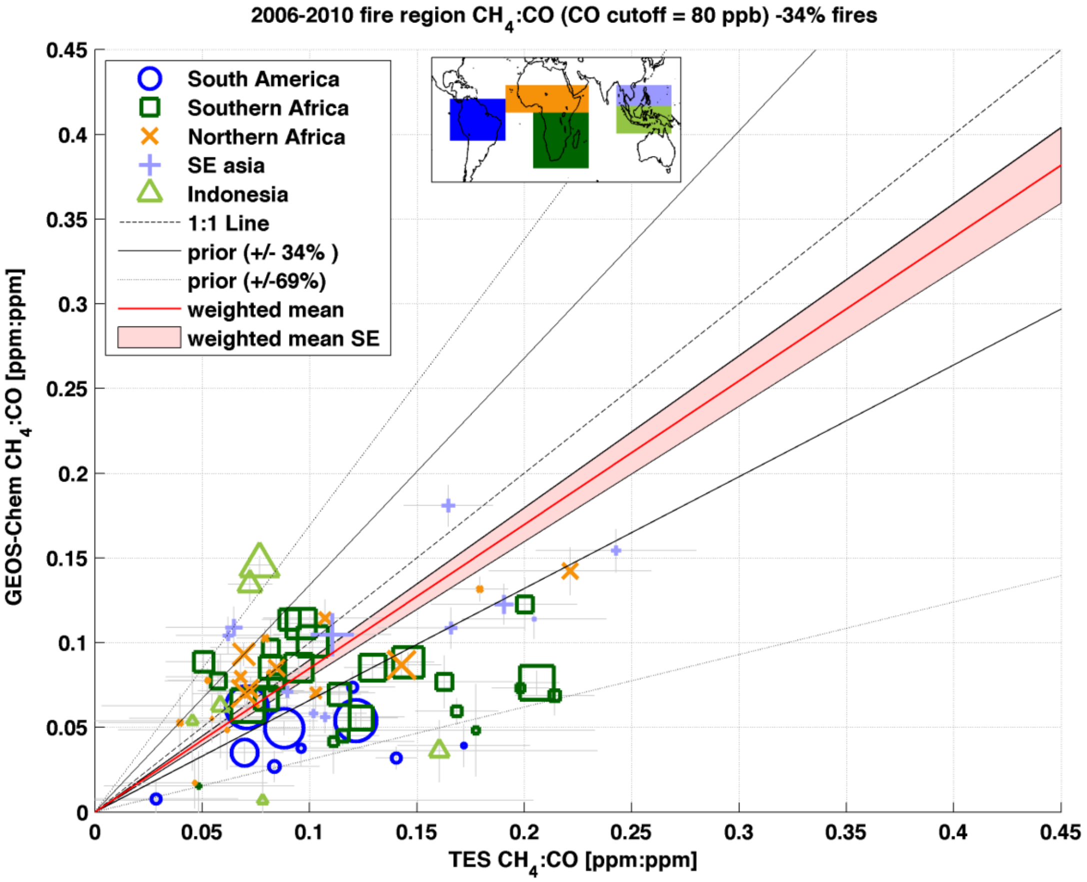

Supplementary Figure 2: Comparison Of CH_4/CO ratios from the GEOS-Chem model and Aura TES data. This figure is the same format as in Figure 6 but the CH_4/CO ratios in the model have been changed by -34%.

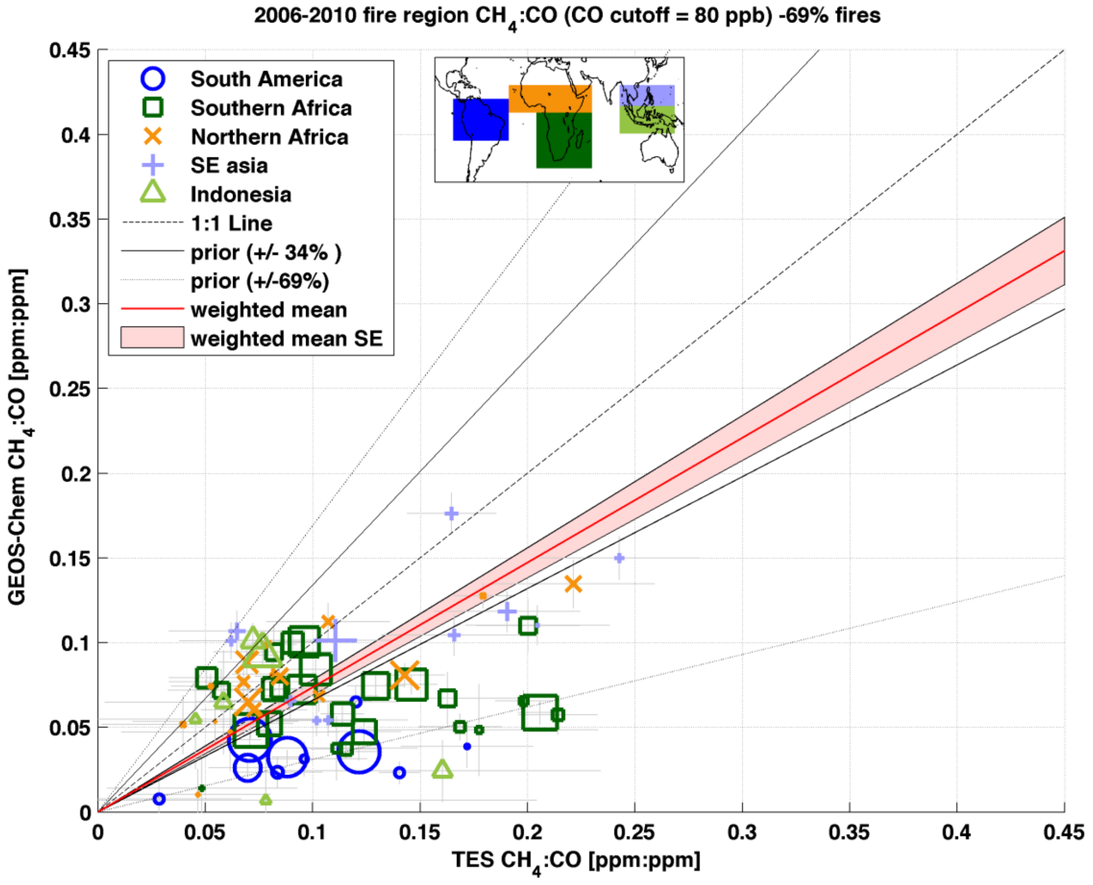

Supplementary Figure 3: Comparison Of CH_4/CO ratios from the GEOS-Chem model and Aura TES data. This figure is the same format as in Figure 6 but the CH_4/CO ratios in the model have been changed by -69%.

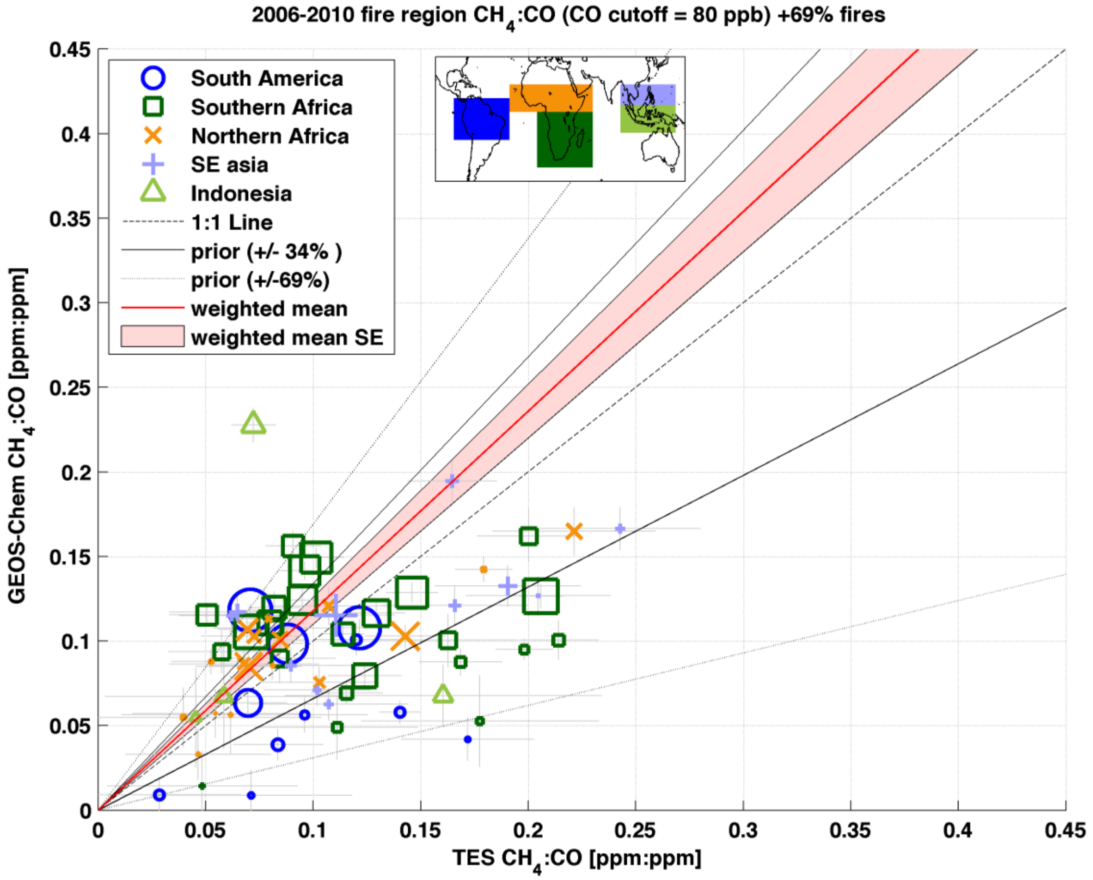

Supplementary Figure 4: Comparison Of CH_4/CO ratios from the GEOS-Chem model and Aura TES data. This figure is the same format as in Figure 6 but the CH_4/CO ratios in the model have been changed by +69%.

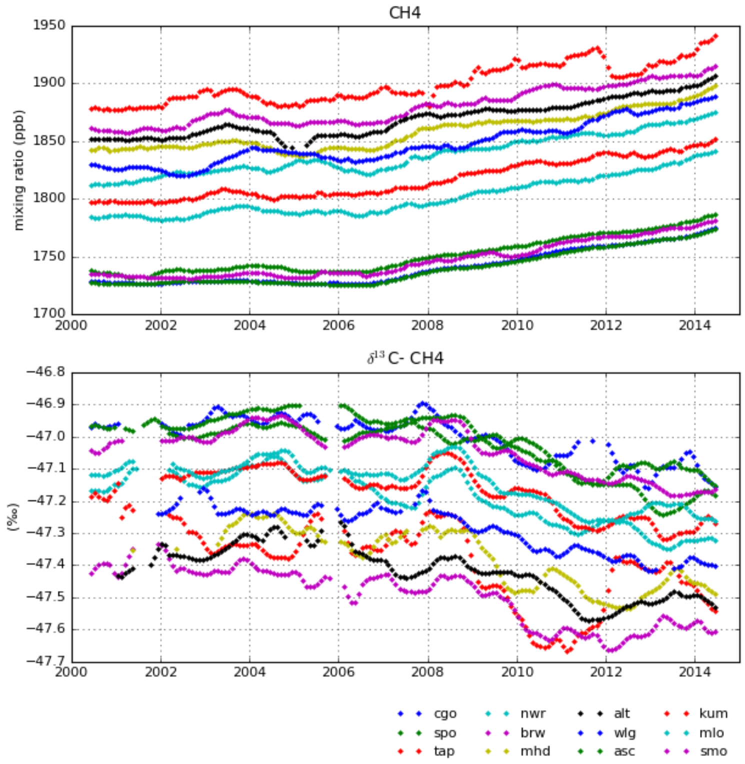

Supplementary Figure 5: CH_4 and $\delta^{13}C$-CH_4 measurements smoothed using a 12-month running mean. (Panel a) CH_4 measurements for all stations used in this analysis. The different colors correspond to the data from the different stations used in this analysis. (Panel b) Similar to panel a but for the corresponding $\delta^{13}C$-CH_4 measurements. The different stations are Ascension island (asc), Tae-ahn (tap), Cape Grim (cgo), Niwot Ridge (nwr), Cape Kumukahi (kum), Barrow (brw), Mauna Loa (mlo), Mace Head (mhd), Tutuila (smo), Alert (alt), the South Pole (spo), and Mt. Waliguan (wlg).

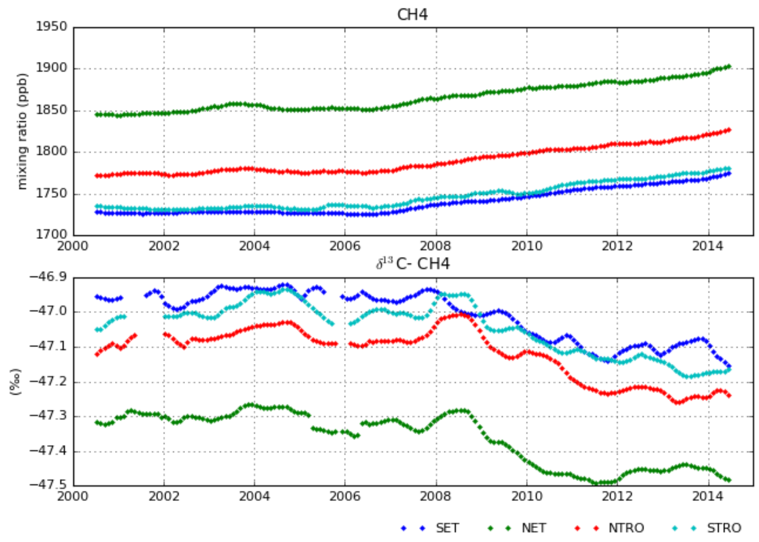

Supplementary Figure 6: Zonal means of the measurements shown in Figure 11.
(Panel a) Zonal means of CH_4. (Panel b) Zonal means of $\delta^{13}C$-CH_4 . NET (Northern Extra Tropics: 30 N to 90 N), NTRO (Northern Tropics: 0 to 30 N), STRO (Southern Tropics: 30 S to 0), SET (Southern Extra Tropics: 90 S to 30 S)

Supplementary Table 1: Assessment of the optimal starting year for the flux inversion time window. The goodness of fit is measured by the RMSD between CH_4 mole fractions measurements and optimized box model simulations for starting years varying between 2005 and 2008. The corresponding strength of the optimized methane emission perturbation is also given.

Years of perturbation	RMSD (ppb)	Strength of emission perturbation (TgCH4/yr)
2005	5.0	18.32
2007	1.7	24.7
2006	2.8	21.2
2008	4.0	28.4
